# Current virtual reality-based rehabilitation interventions in neuro-developmental disorders at developmental ages

**DOI:** 10.3389/fnbeh.2024.1441615

**Published:** 2025-01-15

**Authors:** Micaela Capobianco, Concetto Puzzo, Chiara Di Matteo, Alberto Costa, Walter Adriani

**Affiliations:** ^1^Economic, Psychological and Communication Sciences Department, Niccolò Cusano University, Rome, Italy; ^2^Faculty of Psychology, Università Telematica Internazionale Uninettuno, Rome, Italy; ^3^Center for Behavioural Sciences and Mental Health, Istituto Superiore di Sanità, Rome, Italy; ^4^IRCCS Fondazione Santa Lucia, Rome, Italy

**Keywords:** ADHD, ASD, SLD, neuro-developmental disorders, VR

## Abstract

This mini-review examines the available papers about virtual reality (VR) as a tool for the diagnosis or therapy of neurodevelopmental disorders, focusing on Attention Deficit Hyperactivity Disorder (ADHD), Autism Spectrum Disorder (ASD), and Specific Learning Disorders (SLD). Through a search on literature, we selected 62 studies published between 1998 and 2024. After exclusion criteria, our synoptic table includes 32 studies on ADHD (17 were on diagnostic evaluation and 15 were on therapeutic interventions), 2 on pure ASD, and 2 on pure SLD. These cover a total of 8,139 participants with ADHD (ages 3–19), 458 with ASD (ages 4–19), and 162 with SLD (ages 7–11). Results show that VR offers high ecological validity and enables improvements in cognitive and social skills. Specifically, in individuals with ADHD, VR showed benefits in attention and executive function, with optimal results when combined with pharmacological treatments. For ASD kids, VR proved effective in enhancing social skills and emotional regulation through personalized virtual scenarios. However, the literature on SLD remains limited, suggesting an evolving area of research. Despite limitations related to small sample sizes and technology costs, VR presents a promising outlook for clinical intervention in neuro-developmental disorders, supporting enhanced skills in a safe and controlled environment. We conclude that both immersive and non-immersive VR represents a valuable supplement to traditional therapies, allowing for personalized approaches.

## 1 Introduction

Neurodevelopmental disorders comprise a category of behavioral problems with very different etiologies and characteristics: the complexity of the profile and the degree of severity may vary considerably. In this work we will focus in particular on children and adolescents with specific disease, as Attention Deficit and Hyperactivity Disorder (ADHD), Specific Learning Disorders (SLD), often in comorbidity with ADHD, and Autism Spectrum Disorders (ASD), as far as the use of virtual reality is concerned.

### 1.1 An overview of the symptoms

ADHD is characterized by a difficulty in the domain of attention, that can significantly affect the different contexts of the child’s life: it is present in about 8% of the world’s population with prevalence in male participants (75–80%) compared to girls (20–25%). Inattention subtype was found to be prevalent compared to hyperactivity subtype (Ayano et al., 2023). As illustrated in the studies by Berger et al. (2011) and Cortese et al. (2012), ADHD can progress throughout the life-course. Quite often, individuals with ADHD are sensation seekers who pursue adrenaline-releasing activities, and are vulnerable to substance abuse as well as to behavioral addictions (Davis et al., 2015). The study conducted by Zhao et al. (2019) highlights that participants with ADHD between the ages of 14 and 17 represent an approximately five times-more economic burden on their families than children with typical neurodevelopment. Pang et al. (2021) observed how children with ADHD had greater difficulty following standard school programs, leading to poor academic performance that in turn can lead to learning disabilities (SLD). In various published works between 2006 and 2020 in a wide range of children and adolescents it was shown how there is a comorbidity between ADHD and various behavioral disorders, such as Oppositional Provocative Disorder (ODD), Emotional Disorder (ED); (Coolidge et al., 2000; Berger et al., 2011; Cortese et al., 2012; Vasileva et al., 2020).

In other recent studies, it has been observed that Specific Learning Disabilities (SLD) and Attention Deficit/Hyperactivity Disorder (ADHD) are often present as secondary diagnoses of other primary disorders, on a socio-emotional basis, as happens with Selective Mutism (SM). In children with Selective Mutism, in fact, the clinical inhibition of verbal expression in unfamiliar contexts (and in particular in the school context) would lead over time to the structuring of learning disabilities and behavioral problems such as inattention and hyperactivity (Capobianco and Cerniglia, 2018; Capobianco and Costa, 2024). Specific Learning Disorder (SLD) is characterized by the child or young person’s difficulty in automating learning (Curtin et al., 2019); for this reason, SLD people may have difficulties in different domains, like reading, writing, logical-mathematical performance (Beitchman and Young, 1997). In the DSM-V-TR (APA, 2023) it is noted that patients with SLD may present an impairment in the area of reading with respect to decoding (speed and accuracy) and/or in understanding the meaning of what is read; in the area of writing, with difficulty in spelling and/or handwriting; in the logical mathematical area, with respect to written and mental operations, procedures and arithmetic reasoning. The study by Moll et al. (2014) found a differential gender prevalence, associated with the fact that girls manifested greater problems in arithmetics, while boys manifested greater problems in spelling. With respect to the underlying cognitive processes implicated in SLD, many studies highlight deficits in working memory and in the area of attention (De Simone et al., 2023).

In SLD, working memory is found to be impaired (Chieffo et al., 2023; Martínez-Briones et al., 2020) and comorbidity may occur with developmental dyslexia (Solan, 1993), dysgraphia (Chung et al., 2020) and dyscalculia (Castaldi et al., 2020). In children with typical development and age of 8 to 10 years old, fluency due to phonemic fusion (supported by the phonological store) influences reading speed, consequently facilitating reading comprehension (Orsolini et al., 2023). According to the study by Cristofani et al. (2023) and in agreement with the studies by Toffalini et al. (2018) and Nachshon and Horowitz-Kraus (2018), the predictive factor in individuals with SLD (age 7–18 years) consists of the working memory index affecting processing speed. Another source of difficulties (for learning by kids with SLD) falls in the sphere of executive functions, as if they lack the capability to regulate behavior in new contingencies (Richards et al., 1990; Agostini et al., 2022; Miyake et al., 2000; Diamond, 2013). Sahoo et al. (2015) noted high comorbidity rates among SLD children and ADHD ones, although the latter is not primarily a learning disorder.

The clinical picture of autism spectrum disorder (ASD), with low or high cognitive functioning, is certainly more complex: developmental difficulties fall within secondary disorders. The neurodevelopmental trajectory may also be affected in ASD, defined as the persistent difficulty in interaction, communication, and with stereotypical behaviors that can impair social functioning (APA, 2013; Lord et al., 2018). ASD is present worldwide, not differing significantly between geographic regions (Elsabbagh et al., 2012; Baio et al., 2018), and affects 1 in 59 children (Rylaarsdam and Guemez-Gamboa, 2019), being prevalent in males (three times more) rather than in females (Loomes et al., 2017). A relevant aspect of individuals with autism is the presence of high levels of anxiety and stress (Bozkurt et al., 2019; Cohrs and Leslie, 2017; Fuld, 2018; Makris et al., 2022; De Vaan et al., 2020). In ASD, stereotypical behaviors (Lai et al., 2014) and restricted interests (Bourson and Prevost, 2022) may be concomitant with intellectual disabilities (Xie et al., 2020), disturbed sleep (Devnani and Hegde, 2015), and poor language (Georgiou and Spanoudis, 2021).

In current clinical practice, when a child is suspected of suffering from a neuro-developmental disorder such as ADHD, SLD or ASD, clinicians formulate the diagnosis through a neuropsychological assessment with analogical tools (such as paper questionnaires, structured interviews and standardized tests) and through the clinical observation of the symptoms. Once the diagnosis has been made, the intervention is usually analogical, consisting on the one hand in targeted activities, aimed at rehabilitating the deficient skills; on the other hand, in fostering kid’s inclusion through the involvement of parents (for example, through parent training) and teachers (via observation at school). Clinical and research data suggest that the most functional therapy should be integrated and multidisciplinary, mainly through cognitive-behavioral psychotherapy sessions, speech therapy and psychomotor rehabilitation, with the addition of parent training and regular meetings with parents and teachers if necessary. A cognitive-behavioral intervention strategy (that is widely used and proven to be functional) is certainly the exposure of the patient or family members to problematic situations in everyday life contexts for which it is necessary to modify the resolution strategy, like for Exposure and Response Prevention (ERP) (Law and Boisseau, 2019).

### 1.2 The recent exploitment of VR

With the ongoing digital revolution, children are immersed in highly technological and high-speed contexts: they receive smartphones or tablets as early as 2 years old, and in 5–10 years from now they will come into contact with artificial intelligence. Virtual environment (or Virtual Reality, VR) is a digital, computer-generated, three-dimensional experiential environment. Unlike traditional interfaces (that only allow the user to view a screen), VR allows the user to immerse in an experience, and interact with a tridimensional (3D) world that may simulate or completely differ from the real world. “Immersive” virtual reality is an advanced form of VR that aims to recreate a digital environment through the use of special devices, such as VR viewers, headsets, sensory gloves and motion-tracking sensors. Thus, “non-immersive” virtual reality is a simpler condition: the child simply stands in front of a device which acts as a window to the three-dimensional world. VR, by its own peculiarities, could (1) increase the opportunities for exposure to everyday reality situations through immersive and non-immersive simulation, and (2) challenge the patient on various social and cognitive (Beidel et al., 2021) as well as emotional skills (Gall et al., 2021), through the development of mediated sensation (Botvinick and Cohen, 1998).

“Non-immersive” virtual reality is certainly less expensive and may be easier to apply in the clinical and school setting. “Immersive” virtual reality is more expensive and more challenging with respect to the tools to be used, but it may be the only way to create exposures closer to an individual’s specific reality. In fact, “immersive” VR allows involvement of patients in alternative activities to classical psychotherapy in presence (Riva et al., 2003), with the possibility of simulating different real situations. These tools are nowadays used more and more frequently, in order to implement exposures to situations requiring to choose a given behavior. Intervention is aimed at desensitizing dysfunctional behaviors in favor of others whereas they are more appropriate. In addition to having evaluative and rehabilitative purposes, virtual reality would enable the learning of new skills useful for specific difficulties in different life contexts (Parsons et al., 2017; Maresca et al., 2022; Zhang et al., 2023). For example, VR could be used as an additional compensatory strategy for educational purposes or be used in clinical settings. “Immersive” virtual reality, by recreating virtual environments which are familiar to the individual affected by these disorders, could increase ecological validity and reduce exposure time and costs.

Several systematic reviews have been published regarding the application of virtual reality with therapy for neurodevelopmental disorders at different ages (Tan et al., 2022; Valentine et al., 2020; Ali et al., 2023; Bailey et al., 2021; Li et al., 2023) and for ADHD by geographic origin (Chaulagain et al., 2023; Tosto et al., 2015; Hulsbosch et al., 2021; Lauder et al., 2022; Salari et al., 2023; Cénat et al., 2022; Thomas et al., 2015; Polanczyk et al., 2007; Willcutt, 2012; Alwardat et al., 2024). Some studies have included phobias (North et al., 1998; Lacey et al., 2022; Botella et al., 2017), emotions (Colombo et al., 2021; Geraets et al., 2021; Andreatta et al., 2023; Chirico and Gaggioli, 2019; Dehghan et al., 2022), social skills (Kourtesis et al., 2023; Dechsling et al., 2021; Zhao et al., 2022), mathematics learning (Su et al., 2022; Calvert et al., 2019; Çevikbaş et al., 2023), daily functioning skills (Yip and Man, 2009; Gourlay et al., 2000; Lee et al., 2023). However, most of the work concerns the use of “non immersive” virtual reality in children with ADHD and ASD. There is limited work on children with SLD and it mostly concerns the use of the devices in the school setting. At present, no significant results are yet available from systematic studies on the use of different virtual realities and on large samples of children with ADHD, SLD, or ASD. Further work is warranted, considering the multiplicity of neuropsychological profiles, associated comorbidities, and gender differences that characterize these neurodevelopmental disorders.

## 2 Main objectives

The aim of this mini review is to examine the state of the art on the use of modern virtual tools for the assessment and/or treatment of SLD, ADHD, as specific developmental disorders, and for the assessment and/or treatment of ASD, as a secondary neurodevelopmental disorder in the absence of intellectual disability. Immersive VR is a digital world where a user can be immersed, wearing a special visor: a new reality totally envelops the user, completely masking the perception (at least visual) of the physical world around him\her. “Non-immersive” Virtual Reality is based on the use of a computer or a video-game console, with a monitor to display and input devices (such as keyboards, and controllers), so that the user remains in some way in interaction with the surrounding physical environment.

The use of a video-game is an excellent example of a non-immersive VR experience. Among the types of “non-immersive reality” in the healthcare sector we also find “telepsychiatry” which consists in the use of devices, such as a PC, to provide assistance to patients even remotely. The innovative and useful aspect of this mini review is that we produced a synoptic table useful for an overall view of the literature on the use of virtual reality. With such table, we aimed at highlighting the main results, strengths and limitations (i.e., still open questions) of the available literature regarding the use of virtual reality in clinical practice. The narrow focus of this mini review is therefore limited to the use of VR in patients with SLD, ADHD, or ASD, albeit with different profiles.

This study contributes to deepening the understanding of how virtual reality can be effective in the assessment and rehabilitation of specific domains in neuro-developmental disorders. Our paper is reflecting how VR could be a practice that integrates with the traditional one in ADHD, SLD and ASD. We gathered specific research on dysfunctional aspects under the perspective of a multimodal and multidisciplinary approach, aimed at structuring a targeted intervention program for the individual’s single profile.

## 3 Methods

### 3.1 Search strings and inclusion\exclusion criteria

We performed parallel searches on PubMed, Web of Science, and Scopus in order to find studies relevant to our research objective. Articles were included if the potential assessment or therapy was explored through the use of virtual reality environments; the declared goal of the article had to be either about the “ASSESSMENT” or about the “INTERVENTION.” The former term includes diagnosis or the possibility to quantify symptoms (or other behavioral aspects) shown by patients; the latter term includes therapeutic purposes, namely educational intervention, or training, or rehabilitation or social intervention. Key words used for search were: “ADHD,” “SLD,” “ASD,” “telemedicine,” and “anxiety,” from the side of the disease; “virtual reality,” “mental health technology,” “treatment,” “intervention” and “rehabilitation” from the side of the approach. We also performed further searches to check for studies not found in the previously mentioned databases.

The search was limited to articles published between January 1, 1998 and January 31, 2024. This range of dates was chosen to include the last 25 years, considering that internet-related technology was scarse before 1998. We also carried out further searches in manual mode (namely, using the same keywords declared by a given paper). This was done with less recent papers: the purpose was to see if that search, conducted by those authors more than 10 years ago, would lead to other literature which was published after their paper until today. However, such approach gave nearly no results, probably because they were centered on technology which was in use more than 10 years ago. Another approach was to conduct manual searches where “ADHD” or “SLD” were replaced by other terms which do represent a specific symptom or comorbid condition: for example, one search exploited “dysthmia” because this symptom presents a 20% of comorbidity with ADHD (Goodman, 2009). We consider this further approach to be able to extend the search (within the aforementioned databases) to different spectra of symptoms, and within pathologies that present a comorbidity with ADHD, SLD or ASD.

Eligibility criteria included English-language publications with the following features: (1) specific articles, systematic reviews, and meta-analytic reviews; (2) explicit mention to prevalence of ADHD and/or prevalence of ASD and/or prevalence of SLD; (3) presence of child and adolescent in the recruited sample. Adult-recruiting studies and/or non-English language studies were not included.

For this present mini-review, a total of 62 papers comprising reviews and original research articles (published between 1998 and 2024, obtained by screening from: PubMed, Web of Science, and Scopus databases) were selected, which underwent further screening (see below). The studies reported in the two synoptic tables comprise a raw total of 8,139 participants with ADHD, with an age range of 3 to 19 years; for ASD, the age range is 4 to 19 years for a total number of 458 participants. Finally, for SLD, a lower number of just 162 participants (between the ages of 7 and 11 years) participated.

### 3.2 “In” and “out” of systematic reviews

From our literature search, both original articles and systematic reviews were found. To deal with the unavoidable overlap, we adopted the following strategy. First, all of the reviews that are listed in the table present bibliographic search criteria that pursue the aim of homogeneity in general methods of article collection. Indeed the search strings of all reviews were using terms such as “virtual reality, virtual non-immersive reality, augmented reality, digital therapy” and “ADHD, executive function, inhibition attention, distractibility.” In this way, the eligibility criteria of review aligned on dealing with the most common VR tools for data acquisition as well as the most common symptoms for the target pathology.

Of course, we found original articles that had been excluded by all the listed reviews. These are somewhat “peculiar” articles, which are based on particular technology systems developed independently by the authors and not common in the rest of literature. The study by Kollins et al. (2020) is an example of this situation: the paper is based on the administration of a video game called AKL-T01 as digital therapy. This game was developed on custom-made, proprietary algorithms, with the aim of improving attention and cognitive control; such paper will hardly be included in any systematic review. The study by Wiguna et al. (2022), similarly, realised a prototype of a new “serious game” with the inclusion of Indonesian cultural content. This “cultural bias” obviously makes this paper not general enough to be included in systematic reviews. Moreover, they used machine learning and AI to extend the algorithm, so that the method somewhat evolved autonomously. Their tool is hardly comparable to other tools proposed for the possible diagnosis of ADHD.

Most of the original articles cited and discussed in the systematic reviews are not present in the table: the exploited digital tools often were commercially available and reproducible in nature. However, we left in the table (as a sort of overlap) a total of six original articles that (despite meeting homogeneity of the listed reviews) were particularly fascinating, since they dealt with the issue of virtual classrooms. These six papers used very similar and widely accepted methodologies, and their findings were confirmed by the systematic reviews themselves.

## 4 Discussion and results

### 4.1 Overview of the literature on ADHD

Numerous studies investigating “non-immersive” VR (Krysta et al., 2017; Gallagher, 2004; Hagi et al., 2023) indicate a growing possibility of managing clinical care through telepsychiatry. The use of different VR devices is becoming increasingly applied at developmental age, not only in the clinical field (through the use of specific rehabilitation software) but also in the educational context. Of note, the use of the IWB (Interactive Whiteboard) in the classroom is increasingly popular for cooperative learning (So et al., 2022; Ramsey et al., 2023; Binder et al., 2022). The use of “non-immersive” VR is certainly useful in school settings, but the different virtual devices are supposed to give their best for rehabilitation purposes (Lee et al., 2020). Most of the work conducted so far on VR concerns children with ADHD.

In the specific context of ADHD, some studies observe that the combination of pharmacological treatments with the use of “non-immersive” VR (such as the use of software that reproduces game-like activities to enhance specific functions) has produced good results. Relevant improvement was observed for key symptoms, such as executive dysfunction (Nigg et al., 2004), motivational dysfunction (Carlson and Tamm, 2000), and motor-planning-self-regulation (Mokobane et al., 2019) as well as variability in response time (Johnson et al., 2007).

Other works that have used “immersive” VR confirm that significant improvements are induced on children with ADHD with respect to behavioral flexibility (Dovis et al., 2015), attention span (Steiner et al., 2014), self-regulation of motor and planning skills (Benzing and Schmidt, 2019; van der Oord et al., 2012). These studies also agree that immersive VR allows for assessment and rehabilitation protocols to be carried out in a safer and more replicable context, compared to the very differing span of real exposures to which children with ADHD may be subjected in their daily lives.

In relation to school environments, we discuss here five “ASSESSMENT” research articles that we found to be particularly noteworthy (Pollak et al., 2009; Adams et al., 2008; Bioulac et al., 2012; Areces et al., 2018; Eom et al., 2019). These five studies in the literature (in bold in Table 1) were based on the formal comparison between a virtual classroom and the real classroom. The virtual classroom environment additionally involved the presence of some rows of desks surrounded by typical classroom furniture (blackboard, doors and a window from which a playground could be observed). There were also distractors of a visual (people entering and leaving the classroom; paper airplanes flying around the classroom) and auditory (whispers, falling pencils, moving chairs) nature. The peculiarity of using VR headsets is that perceptions and sensory information from the real world are reduced to a minimum, allowing the participant to live, fully, in this virtual environment. These immersion models, through the use of tasks focused on attention and inhibition of impulsivity, allowed to uncover different profiles of children with ADHD. It was also discovered that the presence of an avatar (namely a virtual teacher) could have a facilitating effect in the usability of immersive VR and enhance the performance results in the educational context.

**Table 1:** Virtual reality studies at developmental age—papers on ADHD only.

Study	Scope	Specific tools	Disease / range age and N	Geo origin	Outcomes	Limitations
Goharinejad et al. (2022) (Review)	INTERVENTION	VR, AR, MR technologies (Mixed)	ADHD / 4–18 y.o N = 2,378	Spain, Germany, France, Ireland, Romania, New Zealand, USA, Canada, Israel, Iran, Korea, China, Taiwan	These tools improve diagnosis and management of ADHD	The search method is primarily focused on the titles and abstracts of papers
He et al. (2023) (Review)	INTERVENTION	Digital therapies (Non-immersive VR)	ADHD / 4–17 y.o. N = 2,169	UK, Germany, USA, Netherlands, Singapore, France, Iran, Australia, Denmark, Israel, Spain, South Korea, China	Testing the effectiveness of digital therapies in kids with ADHD	Digital therapies are not considered as part of an integrated approach but as replacement therapies
Wong et al. (2023) (Review)	INTERVENTION	Computer-aided training programmes neurofeedback and VR (Mixed)	ADHD / 6–12 y.o. N = 1,410	Spain, Denmark, Netherlands, Germany, Norway, USA, Singapore	Testing the effectiveness of using VR to improve social interaction skills	Insignificant results with doubts about the clinical impact
Bashiri et al. (2017) (Review)	INTERVENTION	VR (Immersive)	ADHD / 6–17 y.o. N = 406	Germany, UK, Canada, USA, Taiwan, Spain, New Zealand, Korea	VR in individuals with ADHD for rehabilitation and developing	Little empirical research to support the benefits attributed to virtual reality
Parsons et al. (2019) (Review)	ASSESSMENT	Computerised continuous performance testing (CPT) in three-dimensional virtual (Mixed)	ADHD / 9–16 y.o. N = 362	Canada, Israel, Ireland, Germany, USA, Spain	Clinical comparison of ADHD children’s attention in virtual classrooms	High heterogeneity in results across different tests
Bemanalizadeh et al. (2021) (Review)	INTERVENTION	Telemedicine (Mixed)	ADHD / 3–17 y.o. N = 358	USA, Netherlands, Canada, Germany, Sweden, Singapore	Tele-medicine for ADHD, expanding the therapeutic options	Unclear risk of bias in almost all included studies
Kollins et al. (2020)	INTERVENTION	AKL-T01 (Non-immersive VR)	ADHD / 8–12 y.o. N = 180	USA	Digital therapy for attention and cognitive control	Effects related to treatment of frustration and headaches
Corrigan et al. (2023) (Review)	INTERVENTION	VR (Immersive)	ADHD / 7–18 y.o N = 164	China, USA, South Korea, Iran	VR for global cognitive functioning, attention, and memory	Poor quality of the studies which could affect its validity
Wong et al. (2023)	INTERVENTION	VR (Immersive)	ADHD / 6–12 y.o. N = 90	China	VR in ADHD who find it difficult to navigate real-world social settings	The results in Hong Kong may not be applicable to other settings or populations
Skalski et al. (2021)	ASSESSMENT	VR (Non-immersive VR)	ADHD / 9–15 y.o. N = 87	Poland	Use of VR in biofeedback, for engagement and motivation	The effect of other factors, such as children’s home or school environments, is unclear
Bioulac et al. (2018)	INTERVENTION	Cognitive Recovery VR vs. Drug (Immersive)	ADHD / 7–11 y.o. N = 51	France	VR as therapeutic tool, compared to pharmacological treatment	Sample size not sufficient to generalize to the entire ADHD population
Areces et al. (2018)	**ASSESSMENT**	**VR** (Immersive)	**ADHD / 6–16 y.o.** **N = 50**	**Spain**	**Analysis of difficulties experienced by kids with ADHD**	**Gender imbalance in the sample, with male predominance**
Mangalmurti et al. (2020)	INTERVENTION	VR (Immersive)	ADHD / 6–12 y.o. N = 45	USA	VR and decision-making in individuals with ADHD	The sample is relatively small, which could affect generalization of the results
Seesjärvi et al. (2021)	ASSESSMENT	VR (Immersive)	ADHD / 9–12 y.o. N = 38	Finland	VR, as a alternative methodology	The sample size restricts the selection of analytic techniques
Yeh et al. (2020)	ASSESSMENT	VR (Immersive)	ADHD / 6–16 y.o. N = 37	Taiwan	VR as free alternative to more traditional techniques	The sample size is relatively small
Merzon et al. (2022)	ASSESSMENT	VR (Immersive)	ADHD / 9–13 y.o. N = 37	Finland	VR viewed as non invasive for ADHD participants	A major concern is generalization of results
Rodrigo-Yanguas et al. (2021)	INTERVENTION	VR (Immersive)	ADHD / 12–22 y.o. N = 37	Spain	VR use increased engagement and motivation of children	Few information on ADHD, has shown potential in treating a number of mental diseases.
Bioulac et al. (2012)	**ASSESSMENT**	**VR** (Immersive)	**ADHD / 7–10 y.o.** **N = 36**	**France**	**Children with ADHD can be tested on their capacity to maintain performances over time**	**Relatively small sample size could influence the generalization to a larger population**
Wong et al. (2023)	ASSESSMENT	VR (Immersive)	ADHD / 6–12 y.o. N = 25	China	VR allows therapeutic experiences that can be more effective than traditional methods	These VR systems can be expensive and may not be readily available in all clinical or educational settings
Jang et al. (2021)	INTERVENTION	Combining VR neuro-psychological testing with functional near-infrared spectroscopy (Non-immersive VR)	ADHD / 7–16 y.o. N = 23	South Korea	VR and fNIRS as effective method to evaluate the effects of drug medication	Small number of ADHD participants and absence of a control group
Hong et al. (2021)	ASSESSMENT	VR (Immersive)	ADHD / 9–17 y.o. N = 21	South Korea	VR is a effective tool for studying impact of distractors on ADHD kids	The sample size is small, thus limiting generalization to a larger population
Stokes et al. (2022)	ASSESSMENT	VR (Immersive)	ADHD / 8–12 y.o. N = 20	USA	Precision offered by VR could lead to a deeper understanding of mechanism underlying ADHD	VR systems can be expensive and may not be readily available in all clinical or educational settings
Romero-Ayuso et al. (2021) (Review)	INTERVENTION	VR (Immersive)	ADHD / 8–18 y.o. N = 20	South Korea, USA	Comprehensive and objective view of the effectiveness of VR-based interventions for ADHD	Variability found in the study designs and methodologies across papers included in the review
Pollak et al. (2009)	**ASSESSMENT**	**Test of continuous performance (CPT) in VR environment** (Immersive)	**ADHD / 9–17 y.o.** **N = 20**	**Israel**	**The use of VR in CPT creates a more dynamic testing environment that could better reflect real-world contexts**	**Lack of comparison with traditional assessment methods, limiting the effectiveness and reliability**
Eom et al. (2019)	**ASSESSMENT**	**VR** (Non-immersive VR)	**ADHD / 6–17 y.o.** **N = 20**	**South Korea**	**VR-CPT can accurately reflect the real-life attentional behaviors of children with ADHD**	**No assessment methods to draw comparison**
Adams et al. (2008)	**ASSESSMENT**	**VR** (Immersive)	**ADHD / 8–14 y.o.** **N = 19**	**USA**	**Increased inattention in children with ADHD using VR compared to normotype individuals**	**Children with ADHD are more susceptible to distractions in the VR environment**
Shema-Shiratzky et al. (2018)	INTERVENTION	VR (Non-immersive VR)	ADHD / 8–12 y.o. N = 14	Israel	A cognitive stimulation through VR, to improve the cognitive function	The sample size is relatively small
Lim et al., 2020	INTERVENTION	Cognitive training (Mixed)	ADHD / 5–17 y.o. N = 12	Singapore	Analysis of increased access to care and the possibility of rapid early intervention	Heterogeneity of results
Parsons et al. (2007)	**ASSESSMENT**	**VR** (Immersive)	**ADHD / 8–12 y.o.** **N = 10**	**USA**	**The VR classroom can be a valid tool to accurately assess attention performance**	**A major concern is very small sample size in each group**
Shiri et al. (2014)	ASSESSMENT	VR (Mixed)	ADHD / Not specified	Israel	VR in ADHD children could help them cope better with daily challenges	A small sample size in each group
Wiguna et al. (2022)	ASSESSMENT	VR and machine learning (Immersive)	ADHD / Not specified	Indonesia	VR could be providing a stimulating context for assessing behaviors by offering a unique perspective	Advanced VR systems algorithms require significant investment
Wiguna et al. (2020)	ASSESSMENT	VR and learning models (deep learning) (Immersive)	ADHD / Not specified	Indonesia	Combination of virtual reality with deep learning	It needs further validation of effectiveness and accuracy in real-world diagnosis of ADHD

VR, virtual reality.

The sixth “ASSESSMENT” study (sixth in bold in Table 1) by Parsons et al. (2007) similarly exploited the virtual classroom in which elements (found in the real school in everyday life) were proposed as an ecologically valid environment. In particular, the avatar showed a combined effectiveness between verbal instructions and perceived physical proximity (of the virtual teacher). Data were showing quite clearly that omissions, hyperactivity and impulsivity were significantly associated with individuals diagnosed with ADHD, discriminating them from children with sufficient levels of attention. This highlights how the school environment inserted in a virtual context can be a valid tool to analyze the various behavioral components underlying ADHD. Furthermore, such kind of virtual school seems to facilitate the development of positive emotions and motivation in the child, thus improving performance in the execution of tasks and decreasing the level of anxiety (Bioulac et al., 2012).

The studies by Goharinejad et al. (2022) and He et al. (2023) highlighted the importance of digital technologies (immersive, non-immersive and augmented reality) both for improving the diagnosis of ADHD and for reducing inattention, in agreement with several studies (Kollins et al., 2020; Yeh et al., 2020; Hong et al., 2021; Stokes et al., 2022; Romero-Ayuso et al., 2021). Wong and Qin (2023) highlighted how the VR intervention was able to qualitatively improve the capacity for social interaction. This, in accordance with the study by Bashiri et al. (2017) which determines how VR is able to provide optimal support for rehabilitation in children with ADHD (Wong et al., 2023). In other words, as highlighted by the study by Parsons et al. (2019), the application of VR has allowed to observe how social virtual environments (i.e., school classes) improve the concept of ecological validity (Seesjärvi et al., 2021; Parsons et al., 2007), allowing the evaluation of the (possible) effects of drugs in individual who use simulation vectors (Jang et al., 2021). A further domain of the virtual impact on children with ADHD was explored by the study by Bemanalizadeh et al. (2021) where it is observed how telemedicine (virtual reality classrooms, games or videoconferencing) can be useful for therapeutic improvement, in accordance with the study by Lim et al., 2020. A relevant study (Merzon et al., 2022) has highlighted how the use of a model with eye tracker can be resolved as an accurate means for the prediction of ADHD. The improvement of cognitive functioning (Corrigan et al., 2023; Shema-Shiratzky et al., 2018) and motivation (Skalski et al., 2021; Rodrigo-Yanguas et al., 2021) could demonstrate how the immersive environment can be useful to individuals with ADHD (Wong and Qin, 2023; Mangalmurti et al., 2020; Bioulac et al., 2012) also through the emotionally positive experience (Shiri et al., 2014).

The overall result of the selected works in the field of ADHD indicates how an integrated treatment, also carried out through VR, could certainly be more functional both in the evaluation phase and for the rehabilitation of children with ADHD. The use of “immersive” VR allows for greater replicability of therapeutic situations aimed at enhancing problem-solving skills, and therefore offers the possibility of more easily generalizing the results of the intervention.

### 4.2 Much less literature was found on SLD and ASD

Children with ADHD often have learning problems, and generally a SLD is closely related to attention difficulties, especially at school. Through our literature search, however, only a very limited number of studies were found on the application of VR (both immersive and non-immersive) in individuals with SLD in comorbidity with ADHD (see Table 2).

**Table 2:** Virtual reality studies at developmental age—papers not on ADHD only.

Study	Specific tools	Disease / range age and N	Geo origin	Outcomes	Limitations
Mesa-Gresa et al. (2018)	VR (Mixed)	ASD / 5–15 y.o. N = 398	China, India, Indonesia, Denmark, USA, Taiwan, Spain, UK, Australia	Improved communication and social skills	The relatively small sample size may limit generalizability of the results
Frolli et al. (2022)	VR (Non-immersive VR)	ASD / 9–10 y.o. N = 60	Italy	VR in children with ASD as facilitating rapid learning and complex social skills	Many of the studies lack validations, which poses challenges in confirming the efficacy of VR
Satu et al. (2023)	VR (Mixed)	ADHD & ASD /ADHD N = 32 ASD N = 111	Netherlands, UK, Australia, Norway, USA	Understanding how VR might influence individual and social functioning	It needs further research to strengthen the effectiveness of VR
Boo et al. (2021)	VR (Immersive)	ADHD & ASD / 8–16 y.o. N = 76	USA	The VR paradigm could be an innovation over traditional method	It is impossible to determine if the VR job triggers cognitive, social, or a combination of demands.
Wang and Reid (2010)	VR (Mixed)	ADHD, ASD & cerebral palsy / 4–18 y.o. ADHD N = 20 ASD N = 9	Canada	VR could be an improvement for ADHD, autism and cerebral palsy	Relationship between the social and cognitive demands has not been explored in detail
Parsons et al. (2017)	VR (Non-immersive VR)	Anxiety, ASD, PTSD and ADHD / 4–17 y.o. N = Not specified	USA	VR can create simulated environments that offer new opportunities for smarter intervention	The availability and cost of VR may be prohibitive.
Köse et al. (2022)	Therapist guided game-based intervention (Non-immersive VR)	SLD / 7–10 y.o. N = 138	Turkey	Controlled trial on visual perception skills in children with SLD	Accessibility and cost issues (see above)
Di Giusto et al. (2023)	VR (Non-immersive VR)	SLD / 7–11 y.o. N = 24	Italy	A study on executive functions improved by VR based intervention	Lack of a control group. Lack of an academic skills evaluation. Impossible to measure any advances more precisely.

VR, virtual reality.

Studies on children with SLD alone concern not only application of non-immersive VR of different types, for example tablets or specific software, but also the mediation of the electronic whiteboard (IWB) in the classroom. Some works have investigated how non-immersive VR can be functional on the individual aspects that characterize SLD, such as executive functions (Livesey et al., 2006), cognitive flexibility (Cartwright et al., 2019), difficulties in reading (Cano et al., 2021) and in the logical-mathematical area (de Castro et al., 2014). The studies by Bryant et al. (2019) and by Coelho et al. (2023) highlight that non-immersive VR can help the inclusion of individuals with SLD in real classroom life and in school activities: thus, promoting the development not only of specific skills related to learning but also of the processes underlying teaching activities. The study by Di Giusto et al. (2023) found an increase in visual attention and task-planning skills in the long term, namely over 6 months from the administration of the non-immersive VR. The results extrapolated from the selected works allow to hypothesize that VR could improve the autonomous learning skills of children with SLD and, above all, be also a valid support to be used both at home and at school as an additional compensatory strategy.

Specifically, the study by Frolli et al. (2022) compares two types of intervention for the improvement of social skills: emotional training obtained through the use of VR was formally compared in a parallel-group with traditional emotional training performed individually with a therapist. Results show that both types of intervention displayed the same final recovery for the recognition of primary emotions; however, the group using VR showed shorter acquisition times. An improvement in emotional modulation was also observed by Didehbani et al. (2016) and by Kim and Kim, 2020, which compared elements aimed at the recognition of facial expressions, gestures (Cai et al., 2013) and voice (Golan and Baron-Cohen, 2006). In particular, the study by Didehbani et al. (2016) investigated the impact of a VR Social Cognition Training to enhance social skills in children with ASD between the ages of 7 and 16. Three primary domains were measured pre vs. post intervention: emotion recognition, social attribution, attentional and executive function. Results revealed improvements on all measures with a positive overall effect on analogical reasoning. These studies suggest that the use of a VR platform offers an effective treatment option for improving social impairments commonly found in ASD. VR in general may be a promising, dynamic and effective practice for supporting the basic and complex social skills of these individuals.

In other words, it is highlighted that the use of VR can improve cognitive development (Zhao et al., 2022), social interaction (Miller et al., 2020), spatial cognition (De Luca et al., 2019) and task learning (Shahmoradi and Rezayi, 2022) in children with ASD. For example, two different cognitive trainings were administered by De Luca et al. (2019): the classical cognitive behavioral therapy (CBT) was combined (or not) with an approach using VR called “Nirvana System.” This is a medical device that uses immersive virtual-reality techniques for motor and cognitive neuro-rehabilitation of patients. Only the combined approach provided an improvement in attention processes and in spatial-cognition skills, with a significant reduction of ideomotor stereotypes. According to this experience, the use of VR in addition to CBT could be a useful and promising tool to improve cognitive function in individuals severely affected by ASD.

From the results of all these studies, it emerges that customizable environments in VR can be an additional methodology (complementary to the traditional one) that can lead to greater benefits to daily life in social, emotional and adaptation skills (Yuan and Ip, 2018; Amat et al., 2021). In related neuro-developmental diseases, characterized by both social and communication impairment, classical symptomatic treatment is similarly observed to be more beneficial if VR is also applied (Mesa-Gresa et al., 2018; Satu et al., 2023). Collectively, all these papers do introduce an innovative method (Boo et al., 2021) in the clinical practice.

### 4.3 Current research limitations

One limitation of our study is the small number of items analyzed, especially for SLD. It would be interesting to verify, with a larger sample in the future, the relationship between male and female individuals as well as whether neuro-developmental disorders relate to average IQ. In addition, future analyses could be able to observe whether individuals respond in an improved mode subsequently to the use of customizable virtual environments, compared to people using VR in a static mode.

Relative to individuals with ADHD, the majority of studies were focused on the American social setting, and therefore a meta-analysis including the world-wide view (with other geographical targets such as European, Asian, and African macro areas) would be worth considering in the future. Since the current studies are directed more to the male than the female population with ADHD, it will be necessary to investigate what happens in the female sphere. It can easily be foreseen that girls with ADHD would need a gender-specific, tailored VR approach.

A conceptual limitation is that there are very few published papers related to anatomical studies and interconnected brain structures, at least for the female compared to the male gender. A further limitation is the lack of studies through fMRI or fNIRS in the pre- and post-treatment windows, which could tell us whether there are detectable changes in brain networks. As far as technological engineering is concerned, it could be relatively easy to integrate a device to show images at the level of the eyes with a small fNIRS scanner located at the level of the prefrontal cortex. Hence, data on brain substrates behind selective attention and the decision-making process could be collected in perfect synchrony with the VR task that is being proposed.

With a few exceptions, the studies here reviewed found a low number of participants involved, with an average of 40 participants per original paper: this may have been the result of the different recruitment strategies applied. Of note, the majority of papers addressing ADHD, with a scope of ASSESSMENT, recruited quite few patients (14 out of 17 papers were with *n* < 40); this is really a small sample. Unfortunately, many research groups cannot exploit big and efficient infrastructures, rather they can rely on small clinical entities that receive around 10–20 patients per week. As a matter of fact, the recruitment of such small samples often requires 1 year of work or more. Such a limitation can only be remedied by means of multi-centric recruitment; however, in real settings, large multi-centric studies are expensive and hardly get financed.

Of note, those papers with the lower number of patients aimed to create a school-like setting, with virtual classrooms. Given the average dimension of school classrooms (20 to 30 pupils with 1 to 5 affected by ADHD/ASD), the putative beneficial effect of VR has to be quite great, otherwise it would be negligible by teachers and by peers. Given this premise, a great-enough effect could be seen (and be statistically significant) even on a small recruited sample.

## 5 Concluding remarks: clinical and\or research considerations

The aim of this mini review was to investigate the different ways in which VR has been used so far, for the assessment and\or intervention in some neuro-developmental disorders. We focused on kids with either ADHD, SLD, or ASD. Studies involving individuals with ADHD and SLD have analyzed mainly age groups between 6 and 18 years old, since both diseases manifest clearly at school age (Colomer et al., 2017). Results of the selected works highlight multiple and significant advantages in the use of both “non-immersive” and “immersive” VR.

Numerous studies (Krysta et al., 2017; Gallagher, 2004; Hagi et al., 2023) indicate an improvement in the quality of care through telemedicine (remote therapy, when it is not possible in person). Use of “non-immersive” VR is becoming increasingly applied at developmental age in both the school and the clinical rehabilitation context. The use of different devices allows for the creation of diversified environments that are more engaging and motivating for the child, compared to the exclusive use of traditional methodologies (So et al., 2022; Ramsey et al., 2023; Schueller et al., 2017). The “non-immersive” VR, in fact, seems to be very functional at school and in clinical rehabilitation; it may well be seen as a “compensatory” strategy for children with reduced attention spans and specific difficulties in learning, such as those found in ADHD and SLD. For children with ASD, the use of “non-immersive” VR is rather found to be a complementary strategy when added to cognitive-behavioral practices.

As highlighted by several works, we can state that the use of “non-immersive” VR can optimize clinical and inclusion objectives at school, mostly when used together with traditional methodologies (Carlson and Tamm, 2000; Nigg et al., 2004; Dovis et al., 2015; Mokobane et al., 2019; Benzing and Schmidt, 2019). We can deduce that an integrated approach (consisting of cognitive-behavioral psycho-therapy, speech therapy, psychomotor-skill training) can certainly be functional also if carried out through VR. In the first clinical phase (evaluating the characteristics of children with ADHD, SLD or ASD), VR allows to outline more clearly the specific neuropsychological profile of a child, turning out to be useful for planning a targeted and personalized intervention protocol. Another advantage of using “non-immersive” VR, in parallel with more traditional practices, concerns the increased motivation of the kids together with a decreased frustration and emotional distress, due to lower performance anxiety (Bioulac et al., 2012).

The peculiarity of “immersive” VR is to allow the participant to fully live in another reality that is intended to be created in a safer context and a more replicable way. Compared to traditional strategies, the use of immersive VR allows (1) greater replicability of situations and therefore the possibility of more easily generalizing the results; (2) to intervene on problem-solving skills, hence allowing better results in everyday life (Yuan and Ip, 2018; Amat et al., 2021). Children with ADHD, SLD and ASD present multiple profiles, very different from each other; therefore, the integrated use of immersive and/or non-immersive VR meets the need to prepare personalized intervention protocols for each individual case.

The bibliographic search conducted for this mini-review highlighted that, while there are several studies on the use of immersive and non-immersive VR in ADHD, studies on children with SLD and ASD are still few; those available primarily concern “non-immersive” VR, in the school context. Studies on “immersive” VR are still limited. A greater number of studies with larger samples would be necessary to systematically address the impact of virtual exposures onto problem-solving social skills, specific to the individual children with any neuro-developmental disorder other than ADHD.

Empirical and clinical data show that children with ADHD, SLD and ASD often present with complex conditions characterized by multiple comorbidities. For example, children with ADHD often have a SLD but may also present other associated psychopathologies such as anxiety disorders, social phobia, obsessive-compulsive disorder, selective mutism. The collection of normative data on the effectiveness of different types of VR, on clinical samples distinguished by gender, age and specific comorbidities within a neuro-developmental disorder, could be very important to structure more targeted and personalized rehabilitation protocols for that specific clinical profile.

## References

[ref1] Adams R. Finn P. Moes E. Flannery K. Rizzo A. (2008). Distractibility in attention-deficit/hyperactivity disorder (ADHD): the virtual reality classroom. Child Neuropsychol. 15, 120–135. doi: 10.1080/09297040802169077, PMID: 18608217

[ref2] Agostini F. Zoccolotti P. Casagrande M. (2022). Domain-general cognitive skills in children with mathematical difficulties and dyscalculia: a systematic review of the literature. Brain Sci. 12:239. doi: 10.3390/brainsci12020239, PMID: 35204002 PMC8870543

[ref3] Ali S. G. Wang X. Li P. Jung Y. Bi L. Kim J.. (2023). A systematic review: virtual-reality-based techniques for human exercises and health improvement. Front. Public Health 11:1143947. doi: 10.3389/fpubh.2023.1143947, PMID: 37033028 PMC10076722

[ref4] Alwardat M. Etoom M. Almhdawi K. A. Hawamdeh Z. Khader Y. (2024). Prevalence of attention-deficit hyperactivity disorder in children, adolescents and adults in the Middle East and North Africa region: a systematic review and meta-analysis. BMJ Open 14:e078849. doi: 10.1136/bmjopen-2023-078849, PMID: 38238059 PMC10806616

[ref5] Amat A. Z. Zhao H. Swanson A. Weitlauf A. Warren Z. Sarkar N. (2021). Design of an interactive virtual reality system, INVIRS, for joint attention practice in autistic children. IEEE Trans. Neural Syst. Rehabil. Eng. 29, 1866–1876. doi: 10.1109/tnsre.2021.3108351, PMID: 34460376 PMC8482411

[ref6] Andreatta M. Winkler M. H. Collins P. Gromer D. Gall D. Pauli P.. (2023). VR for studying the neuroscience of emotional responses. Curr. Top. Behav. Neurosci. 65, 161–187. doi: 10.1007/7854_2022_405 36592276

[ref7] APA (2013). Diagnostic and statistical manual of mental disorders: DSM-5. Washington, DC: American Psychiatric Association.

[ref8] APA (2023). Diagnostic and statistical manual of mental disorders: DSM-5-TR™. Washington, DC: American Psychiatric Publishing.

[ref9] Areces D. Dockrell J. García T. González-Castro P. Rodríguez C. (2018). Analysis of cognitive and attentional profiles in children with and without ADHD using an innovative virtual reality tool. PLoS One 13:e0201039. doi: 10.1371/journal.pone.0201039, PMID: 30110334 PMC6093610

[ref11] Ayano G. Demelash S. Gizachew Y. Tsegay L. Alat R. (2023). The global prevalence of attention deficit hyperactivity disorder in children and adolescents: an umbrella review of meta-analyses. J. Affect. Disord. 339, 860–866. doi: 10.1016/j.jad.2023.07.071, PMID: 37495084

[ref12] Bailey B. Bryant L. Hemsley B. (2021). Virtual reality and augmented reality for children, adolescents, and adults with communication disability and neurodevelopmental disorders: a systematic review. Rev. J. Autism Dev. Disord. 9, 160–183. doi: 10.1007/s40489-020-00230-x, PMID: 39687769

[ref13] Baio J. Wiggins L. D. Christensen D. Maenner M. J. Daniels J. L. Warren Z.. (2018). Prevalence of autism spectrum disorder among children aged 8 years — autism and developmental disabilities monitoring network, 11 sites, United States, 2014. Morb. Mortal. Wkly. Rep. Surveill. Summ. 67, 1–23. doi: 10.15585/mmwr.ss6706a1, PMID: 29701730 PMC5919599

[ref14] Bashiri A. Ghazisaeedi M. Shahmoradi L. (2017). The opportunities of virtual reality in the rehabilitation of children with attention deficit hyperactivity disorder: a literature review. Korean J. Pediatr. 60, 337–343. doi: 10.3345/kjp.2017.60.11.337, PMID: 29234356 PMC5725338

[ref15] Beidel D. C. Tuerk P. W. Spitalnick J. Bowers C. A. Morrison K. (2021). Treating childhood social anxiety disorder with virtual environments and serious games: a randomized trial. Behav. Ther. 52, 1351–1363. doi: 10.1016/j.beth.2021.03.003, PMID: 34656191 PMC8531536

[ref16] Beitchman J. H. Young A. (1997). Learning disorders with a special emphasis on reading disorders: A review of the past 10 years. J. Am. Acad. Child Adolesc. Psychiatry 36, 1020–1032. doi: 10.1097/00004583-199708000-00009, PMID: 9256582

[ref17] Bemanalizadeh M. Yazdi M. Yaghini O. Kelishadi R. (2021). A meta-analysis on the effect of telemedicine on the management of attention deficit and hyperactivity disorder in children and adolescents. J. Telemed. Telecare 30, 31–43. doi: 10.1177/1357633x211045186, PMID: 34633251

[ref18] Benzing V. Schmidt M. (2019). The effect of exergaming on executive functions in children with ADHD: A randomized clinical trial. Scand. J. Med. Sci. Sports 29, 1243–1253. doi: 10.1111/sms.13446, PMID: 31050851

[ref001] Berger I. (2011). Diagnosis of attention deficit hyperactivity disorder: much ado about something. PubMed. 13, 571–574. Available at: https://pubmed.ncbi.nlm.nih.gov/21991721, PMID: 21991721

[ref19] Binder F. P. Pöhlchen D. Zwanzger P. Spoormaker V. I. (2022). Facing your fear in immersive virtual reality: avoidance behavior in specific phobia. Front. Behav. Neurosci. 16:827673. doi: 10.3389/fnbeh.2022.827673, PMID: 35571283 PMC9094686

[ref20] Bioulac S. Lallemand S. Rizzo A. Philip P. Fabrigoule C. Bouvard M. P. (2012). Impact of time on task on ADHD patient’s performances in a virtual classroom. Eur. J. Paediatr. Neurol. 16, 514–521. doi: 10.1016/j.ejpn.2012.01.006, PMID: 22269913

[ref21] Bioulac S. Micoulaud-Franchi J. Maire J. Bouvard M. P. Rizzo A. A. Sagaspe P.. (2018). Virtual remediation versus methylphenidate to improve distractibility in children with ADHD: a controlled randomized clinical trial study. J. Atten. Disord. 24, 326–335. doi: 10.1177/1087054718759751, PMID: 29562853

[ref22] Boo C. Alpers-Leon N. McIntyre N. Mundy P. Naigles L. (2021). Conversation during a virtual reality task reveals new structural language profiles of children with ASD, ADHD, and comorbid symptoms of both. J. Autism Dev. Disord. 52, 2970–2983. doi: 10.1007/s10803-021-05175-6, PMID: 34244916

[ref23] Botella C. Fernández-Álvarez J. Guillén V. García-Palacios A. Baños R. (2017). Recent Progress in virtual reality exposure therapy for phobias: A systematic review. Curr. Psycchiatry Rep. 19:42. doi: 10.1007/s11920-017-0788-4, PMID: 28540594

[ref24] Botvinick M. Cohen J. (1998). Rubber hands ‘feel’ touch that eyes see. Nature 391:756. doi: 10.1038/35784, PMID: 9486643

[ref25] Bourson L. Prevost C. (2022). Characteristics of restricted interests in girls with ASD compared to boys: a systematic review of the literature. Eur. Child Adolesc. Psychiatry 33, 987–1004. doi: 10.1007/s00787-022-01998-5, PMID: 35644857

[ref26] Bozkurt G. Uysal G. Düzkaya D. S. (2019). Examination of care burden and stress coping styles of parents of children with autism spectrum disorder. J. Pediatr. Nurs. 47, 142–147. doi: 10.1016/j.pedn.2019.05.005, PMID: 31146248

[ref27] Bryant L. Brunner M. Hemsley B. (2019). A review of virtual reality technologies in the field of communication disability: implications for practice and research. Disabil. Rehabil. Assist. Technol. 15, 365–372. doi: 10.1080/17483107.2018.1549276, PMID: 30638092

[ref28] Cai Y. Chia N. K. H. Thalmann D. Kee N. K. N. Zheng J. Thalmann N. M. (2013). Design and development of a virtual dolphinarium for children with autism. IEEE Trans. Neural Syst. Rehabil. Eng. 21, 208–217. doi: 10.1109/tnsre.2013.2240700, PMID: 23362251

[ref29] Calvert S. L. Putnam M. M. Aguiar N. R. Ryan R. M. Wright C. A. Liu Y. H. A.. (2019). Young children’s mathematical learning from intelligent characters. Child Dev. 91, 1491–1508. doi: 10.1111/cdev.13341, PMID: 31745971 PMC7818392

[ref30] Cano S. R. Benito V. D. Villaverde V. A. Martín L. M. (2021). Design of a Virtual Reality Software to promote the learning of students with dyslexia. Sustain. For. 13:8425. doi: 10.3390/su13158425

[ref31] Capobianco M. Cerniglia L. (2018). Communicative, cognitive and emotional issues in selective mutism. Interact. Stud. 19, 445–458. doi: 10.1075/is.17018.cap

[ref32] Capobianco M. Costa A. (2024). Selective mutism and comorbidity with specific learning disorders: evaluation and multimodal intervention in a clinical case of a female child from 7 to 11 years of age. Children 11:746. doi: 10.3390/children11060746, PMID: 38929325 PMC11202014

[ref33] Carlson C. L. Tamm L. (2000). Responsiveness of children with attention deficit–hyperactivity disorder to reward and response cost: differential impact on performance and motivation. J. Consult. Clin. Psychol. 68, 73–83. doi: 10.1037/0022-006x.68.1.73, PMID: 10710842

[ref34] Cartwright K. B. Marshall T. R. Huemer C. M. Payne J. B. (2019). Executive function in the classroom: cognitive flexibility supports reading fluency for typical readers and teacher-identified low-achieving readers. Res. Dev. Disabil. 88, 42–52. doi: 10.1016/j.ridd.2019.01.011, PMID: 30851482

[ref35] Castaldi E. Piazza M. Iuculano T. (2020). Learning disabilities: developmental dyscalculia. Handb. Clin. Neurol. 174, 61–75. doi: 10.1016/b978-0-444-64148-9.00005-3 32977896

[ref36] Cénat J. M. Kokou-Kpolou C. K. Blais-Rochette C. Morse C. Vandette M. Dalexis R. D.. (2022). Prevalence of ADHD among black youth compared to White, Latino and Asian youth: a meta-analysis. J. Clin. Child Adolesc. Psychol. 53, 373–388. doi: 10.1080/15374416.2022.2051524, PMID: 35427201

[ref37] Çevikbaş M. Bulut N. Kaiser G. (2023). Exploring the benefits and drawbacks of AR and VR technologies for learners of mathematics: recent developments. Systems 11:244. doi: 10.3390/systems11050244

[ref38] Chaulagain A. Lyhmann I. Halmøy A. Widding-Havneraas T. Nyttingnes O. Bjelland I.. (2023). A systematic meta-review of systematic reviews on attention deficit hyperactivity disorder. Eur. Psychiatry 66:e90. doi: 10.1192/j.eurpsy.2023.2451, PMID: 37974470 PMC10755583

[ref39] Chieffo D. P. R. Arcangeli V. Moriconi F. Marfoli A. Lino F. Vannuccini S.. (2023). Specific learning disorders (SLD) and behavior impairment: comorbidity or specific profile? Children 10:1356. doi: 10.3390/children10081356, PMID: 37628355 PMC10453094

[ref40] Chirico A. Gaggioli A. (2019). When virtual feels real: comparing emotional responses and presence in virtual and natural environments. Cyberpsychol. Behav. Soc. Netw. 22, 220–226. doi: 10.1089/cyber.2018.0393, PMID: 30730222

[ref41] Chung P. J. Patel D. R. Nizami I. (2020). Disorder of written expression and dysgraphia: definition, diagnosis, and management. Transl. Pediatr. 9, S46–S54. doi: 10.21037/tp.2019.11.01, PMID: 32206583 PMC7082241

[ref42] Coelho L. Laska-Leśniewicz A. Pereira E. Sztobryn-Giercuszkiewicz J. (2023). Inclusion and adaptation beyond disability: using virtual reality to foster empathy. Med. Pr. 74, 171–185. doi: 10.13075/mp.5893.01386, PMID: 37695931

[ref43] Cohrs A. Leslie D. (2017). Depression in parents of children diagnosed with autism Spectrum disorder: a claims-based analysis. J. Autism Dev. Disord. 47, 1416–1422. doi: 10.1007/s10803-017-3063-y, PMID: 28214978

[ref44] Colombo D. Díaz-García A. Fernández-Álvarez J. Botella C. (2021). Virtual reality for the enhancement of emotion regulation. Clin. Psychol. Psychother. 28, 519–537. doi: 10.1002/cpp.2618, PMID: 34048621

[ref45] Colomer C. Berenguer C. Roselló B. Baixauli I. Miranda A. (2017). The impact of inattention, hyperactivity/impulsivity symptoms, and executive functions on learning behaviors of children with ADHD. Front. Psychol. 8:540. doi: 10.3389/fpsyg.2017.00540, PMID: 28446885 PMC5388736

[ref46] Coolidge F. L. Thede L. L. Young S. E. (2000). Heritability and the comorbidity of attention deficit hyperactivity disorder with behavioral disorders and executive function deficits: a preliminary investigation. Dev. Neuropsychol. 17, 273–287. doi: 10.1207/s15326942dn1703_1, PMID: 11056845

[ref47] Corrigan N. Păsărelu C. Voinescu A. (2023). Immersive virtual reality for improving cognitive deficits in children with ADHD: a systematic review and meta-analysis. Virtual Real. 27, 3545–3564. doi: 10.1007/s10055-023-00768-1, PMID: 36845650 PMC9938513

[ref48] Cortese S. Kelly C. Chabernaud C. Proal E. Di Martino A. Milham M. P.. (2012). Toward systems neuroscience of ADHD: a meta-analysis of 55 FMRI studies. Am. J. Psychiatry 169, 1038–1055. doi: 10.1176/appi.ajp.2012.11101521, PMID: 22983386 PMC3879048

[ref49] Cristofani P. Lieto M. Casalini C. Pecini C. Baroncini M. Pessina O.. (2023). Specific learning disabilities and emotional-behavioral difficulties: phenotypes and role of the cognitive profile. J. Clin. Med. 12:1882. doi: 10.3390/jcm12051882, PMID: 36902669 PMC10003319

[ref50] Curtin M. Willis D. Enneking B. (2019). Specific learning disabilities: the family physician’s role. Am. Fam. Physician 100, 628–635. Available at: https://pubmed.ncbi.nlm.nih.gov/31730315 31730315

[ref51] Davis C. Cohen A. Davids M. Rabindranath A. (2015). Attention-deficit/hyperactivity disorder in relation to addictive behaviors: A moderated-mediation analysis of personality-risk factors and sex. Front. Psych. 6:47. doi: 10.3389/fpsyt.2015.00047 PMC4403287 25941494

[ref52] De Castro M. V. Bissaco M. A. Panccioni B. M. Rodrigues S. C. M. Domingues A. M. (2014). Effect of a virtual environment on the development of mathematical skills in children with dyscalculia. PLoS One 9:e103354. doi: 10.1371/journal.pone.0103354, PMID: 25068511 PMC4113388

[ref53] De Luca R. Leonardi S. Portaro S. Cause M. L. De Domenico C. Colucci A. P.. (2019). Innovative use of virtual reality in autism spectrum disorder: A case-study. Appl. Neuropsychol. Child 10, 90–100. doi: 10.1080/21622965.2019.1610964, PMID: 31092007

[ref54] De Simone M. S. Costa A. Tieri G. Taglieri S. (2023). The effectiveness of an immersive virtual reality and telemedicine-based cognitive intervention on prospective memory in Parkinson’s disease patients with mild cognitive impairment and healthy aged individuals: design and preliminary baseline results of a placebo-controlled study. Front. Psychol. 14:1268337. doi: 10.3389/fpsyg.2023.1268337 37928597 PMC10622796

[ref55] De Vaan G. Beijers R. Vervloed M. P. J. Knoors H. Bloeming-Wolbrink K. A. De Weerth C.. (2020). Associations between cortisol stress levels and autism symptoms in people with sensory and intellectual disabilities. Front. Educ. 5:540387. doi: 10.3389/feduc.2020.540387, PMID: 39687894

[ref56] Dechsling A. Orm S. Kalandadze T. Sütterlin S. Øien R. A. Shic F.. (2021). Virtual and augmented reality in social skills interventions for individuals with autism spectrum disorder: A scoping review. J. Autism Dev. Disord. 52, 4692–4707. doi: 10.1007/s10803-021-05338-5, PMID: 34783991 PMC9556391

[ref57] Dehghan B. Saeidimehr S. Sayyah M. Rahim F. (2022). The effect of virtual reality on emotional response and symptoms provocation in patients with OCD: A systematic review and Meta-analysis. Front. Psych. 12:733584. doi: 10.3389/fpsyt.2021.733584, PMID: 35177996 PMC8846333

[ref58] Devnani P. Hegde A. U. (2015). Autism and sleep disorders. J. Pediatr. Neurosci. 10, 304–307. doi: 10.4103/1817-1745.174438, PMID: 26962332 PMC4770638

[ref59] Di Giusto V. Purpura G. Zorzi C. F. Blonda R. Brazzoli E. Meriggi P.. (2023). Virtual reality rehabilitation program on executive functions of children with specific learning disorders: a pilot study. Front. Psychol. 14:1241860. doi: 10.3389/fpsyg.2023.1241860, PMID: 37637891 PMC10457143

[ref60] Diamond A. (2013). Executive functions. Annu. Rev. Psychol. 64, 135–168. doi: 10.1146/annurev-psych-113011-143750, PMID: 23020641 PMC4084861

[ref61] Didehbani N. Allen T. T. Kandalaft M. R. Krawczyk D. C. Chapman S. B. (2016). Virtual reality social cognition training for children with high functioning autism. Comput. Hum. Behav. 62, 703–711. doi: 10.1016/j.chb.2016.04.033 PMC3536992 22570145

[ref62] Dovis S. Van Der Oord S. Wiers R. W. Prins P. J. M. (2015). Improving executive functioning in children with ADHD: training multiple executive functions within the context of a computer game. A randomized double-blind placebo controlled trial. PLoS One 10:e0121651. doi: 10.1371/journal.pone.0121651, PMID: 25844638 PMC4386826

[ref63] Elsabbagh M. Divan G. Koh Y. J. Kim Y. S. Kauchali S. Marcín C.. (2012). Global prevalence of autism and other pervasive developmental disorders. Autism Res. 5, 160–179. doi: 10.1002/aur.239, PMID: 22495912 PMC3763210

[ref64] Eom H. Kim K. Lee S. Hong Y. Heo J. Kim J.. (2019). Development of virtual reality continuous performance test utilizing social cues for children and adolescents with attention-deficit/hyperactivity disorder. Cyberpsychol. Behav. Soc. Netw. 22, 198–204. doi: 10.1089/cyber.2018.0377, PMID: 30672714

[ref65] Frolli A. Savarese G. Di Carmine F. Bosco A. Saviano E. Rega A.. (2022). Children on the autism spectrum and the use of virtual reality for supporting social skills. Children 9:181. doi: 10.3390/children9020181, PMID: 35204903 PMC8870236

[ref66] Fuld S. (2018). Autism Spectrum disorder: the impact of stressful and traumatic life events and implications for clinical practice. Clin. Soc. Work. J. 46, 210–219. doi: 10.1007/s10615-018-0649-6, PMID: 30100640 PMC6061115

[ref67] Gall D. Roth D. Stauffert J. Zarges J. Latoschik M. E. (2021). Embodiment in virtual reality intensifies emotional responses to virtual stimuli. Front. Psychol. 12: 674179. doi: 10.3389/fpsyg.2021.674179, PMID: 34552525 PMC8450414

[ref68] Gallagher T. E. (2004). Augmentation of special-needs services and information to students and teachers "ASSIST"--a telehealth innovation providing school-based medical interventions. Hawaii Med. J. 63, 300–309, PMID: 15570717

[ref69] Georgiou N. Spanoudis G. (2021). Developmental language disorder and autism: commonalities and differences on language. Brain Sci. 11:589. doi: 10.3390/brainsci11050589, PMID: 33946615 PMC8147217

[ref70] Geraets C. Tuente S. K. Lestestuiver B. Van Beilen M. Nijman S. A. Marsman J.. (2021). Virtual reality facial emotion recognition in social environments: an eye-tracking study. Internet Interv. 25:100432. doi: 10.1016/j.invent.2021.100432, PMID: 34401391 PMC8350588

[ref72] Goharinejad S. Goharinejad S. Hajesmaeel-Gohari S. Bahaadinbeigy K. (2022). The usefulness of virtual, augmented, and mixed reality technologies in the diagnosis and treatment of attention deficit hyperactivity disorder in children: an overview of relevant studies. BMC Psychiatry 22:4. doi: 10.1186/s12888-021-03632-1, PMID: 34983446 PMC8728980

[ref73] Golan O. Baron-Cohen S. (2006). Systemizing empathy: teaching adults with Asperger syndrome or high-functioning autism to recognize complex emotions using interactive multimedia. Dev. Psychopathol. 18, 591–617. doi: 10.1017/s0954579406060305, PMID: 16600069

[ref74] Goodman D. (2009). Adult ADHD and comorbid depressive disorders: diagnostic challenges and treatment options. CNS Spectr. 14, 5–7. doi: 10.1017/s1092852900024810, PMID: 19773716

[ref75] Gourlay D. Lun K. C. Lee Y. N. Tay J. (2000). Virtual reality for relearning daily living skills. Int. J. Med. Inform. 60, 255–261. doi: 10.1016/s1386-5056(00)00100-3, PMID: 11137470

[ref76] Hagi K. Kurokawa S. Takamiya A. Fujikawa M. Kinoshita S. Iizuka M.. (2023). Telepsychiatry versus face-to-face treatment: systematic review and meta-analysis of randomised controlled trials. Br. J. Psychiatry 223, 407–414. doi: 10.1192/bjp.2023.86, PMID: 37655816 PMC10895502

[ref77] He F. Qi Y. Zhou Y. Cao A. Yue X. Fang S.. (2023). Meta-analysis of the efficacy of digital therapies in children with attention-deficit hyperactivity disorder. Front. Psych. 14:1054831. doi: 10.3389/fpsyt.2023.1054831, PMID: 37260755 PMC10228751

[ref78] Hong N. Kim J. Kwon J. Eom H. Kim E. (2021). Effect of distractors on sustained attention and hyperactivity in youth with attention deficit hyperactivity disorder using a mobile virtual reality school program. J. Atten. Disord. 26, 358–369. doi: 10.1177/1087054720986229, PMID: 33430697

[ref79] Hulsbosch A. De Meyer H. Beckers T. Danckaerts M. Van Liefferinge D. Tripp G.. (2021). Systematic review: attention-deficit/hyperactivity disorder and instrumental learning. J. Am. Acad. Child Adolesc. Psychiatry 60, 1367–1381. doi: 10.1016/j.jaac.2021.03.009, PMID: 33862167

[ref80] Jang S. Choi J. Oh J. Yeom J. Hong N. Lee N.. (2021). Use of virtual reality working memory task and functional near-infrared spectroscopy to assess brain hemodynamic responses to methylphenidate in ADHD children. Front. Psych. 11:564618. doi: 10.3389/fpsyt.2020.564618, PMID: 33551860 PMC7859615

[ref81] Johnson K. Kelly S. P. Bellgrove M. A. Barry E. Cox M. Gill M.. (2007). Response variability in attention deficit hyperactivity disorder: evidence for neuropsychological heterogeneity. Neuropsychologia 45, 630–638. doi: 10.1016/j.neuropsychologia.2006.03.034, PMID: 17157885

[ref82] Kim S. Kim E. (2020). The use of virtual reality in psychiatry: A review. J. Korean Acad. Child Adolesc. Psychiatry 31, 26–32. doi: 10.5765/jkacap.190037, PMID: 32612410 PMC7324842

[ref83] Kollins S. H. DeLoss D. J. Cañadas E. Lutz J. Findling R. L. Keefe R. S. E.. (2020). A novel digital intervention for actively reducing severity of paediatric ADHD (STARS-ADHD): a randomised controlled trial. Lancet Digital Health 2, e168–e178. doi: 10.1016/s2589-7500(20)30017-0, PMID: 33334505

[ref84] Köse B. Temizkan E. Aran O. T. Galipoğlu H. Torpil B. Pekçetin S.. (2022). Where exactly is the therapist in virtual reality and game-based rehabilitation applications? A randomized controlled trial in children with specific learning disability. Games Health J. 11, 200–206. doi: 10.1089/g4h.2021.0241, PMID: 35666260

[ref85] Kourtesis P. Kouklari E. Ρούσσος Π. Mantas V. Papanikolaou Κ. Skaloumbakas C.. (2023). Virtual reality training of social skills in adults with autism Spectrum disorder: an examination of acceptability, usability, user experience, social skills, and executive functions. Behav. Sci. 13:336. doi: 10.3390/bs13040336, PMID: 37102850 PMC10136366

[ref86] Krysta K. Krzystanek M. Cubała W. J. Wiglusz M. S. Jakuszkowiak-Wojten K. Gałuszko-Węgielnik M.. (2017). Telepsychiatry and virtual reality in the Treatment of patients with intellectual and developmental disabilities. Psychiatr. Danub. 29, 656–659. Available at: https://pubmed.ncbi.nlm.nih.gov/28953847 28953847

[ref87] Lacey C. Frampton C. Beaglehole B. (2022). oVRcome – self-guided virtual reality for specific phobias: A randomised controlled trial. Aust. N. Z. J. Psychiatry 57, 736–744. doi: 10.1177/00048674221110779, PMID: 35818296

[ref88] Lai M. Lombardo M. Baron-Cohen S. (2014). Autism. Lancet 383, 896–910. doi: 10.1016/s0140-6736(13)61539-1 24074734

[ref89] Lauder K. McDowall A. Tenenbaum H. R. (2022). A systematic review of interventions to support adults with ADHD at work—implications from the paucity of context-specific research for theory and practice. Front. Psychol. 13:893469. doi: 10.3389/fpsyg.2022.893469, PMID: 36072032 PMC9443814

[ref90] Law C. Boisseau C. L. (2019). Exposure and response prevention in the treatment of obsessive-compulsive disorder: current perspectives. Psychol. Res. Behav. Manag. 12, 1167–1174. doi: 10.2147/prbm.s211117, PMID: 31920413 PMC6935308

[ref91] Lee L. Choi S. Lee H. S. Han S. (2023). Efficacy analysis of virtual reality-based training for activities of daily living and functional task training in stroke patients: A single-subject study. Medicine 102:e33573. doi: 10.1097/md.0000000000033573, PMID: 37083778 PMC10118341

[ref92] Lee H. Lim J. Jeon B. Song C. (2020). Non-immersive virtual reality rehabilitation applied to a task-oriented approach for stroke patients: a randomized controlled trial. Restor. Neurol. Neurosci. 38, 165–172. doi: 10.3233/rnn-190975, PMID: 32176674

[ref93] Li R. C. Belter M. Liu J. Lukosch H. (2023). Immersive virtual reality enabled interventions for autism spectrum disorder: a systematic review and meta-analysis. Electronics 12:2497. doi: 10.3390/electronics12112497

[ref94] Lim C. G. Lim-Ashworth N. S. Fung D. S. (2020). Updates in technology-based interventions for attention deficit hyperactivity disorder. Curr. Opin. Psychiatry 33, 577–585. doi: 10.1097/yco.0000000000000643, PMID: 32858596 PMC7575028

[ref95] Livesey D. J. Keen J. Rouse J. M. White F. A. (2006). The relationship between measures of executive function, motor performance and externalising behaviour in 5- and 6-year-old children. Hum. Mov. Sci. 25, 50–64. doi: 10.1016/j.humov.2005.10.008, PMID: 16442172

[ref96] Loomes R. Hull L. Mandy W. P. L. (2017). What is the male-to-female ratio in autism spectrum disorder? A systematic review and meta-analysis. J. Am. Acad. Child Adolesc. Psychiatry 56, 466–474. doi: 10.1016/j.jaac.2017.03.013, PMID: 28545751

[ref002] Lord C. (2018). ‘Autism spectrum disorder’, The Lancet. 392, 508–520. doi: 10.1016/s0140-6736(18)31129-2, PMID: 30078460 PMC7398158

[ref97] Makris G. Agorastos A. Chrousos G. P. Pervanidou P. (2022). Stress system activation in children and adolescents with autism spectrum disorder. Front. Neurosci. 15:756628. doi: 10.3389/fnins.2021.756628, PMID: 35095389 PMC8793840

[ref98] Mangalmurti A. Kistler W. D. Quarrie B. Sharp W. Persky S. Shaw P. (2020). Using virtual reality to define the mechanisms linking symptoms with cognitive deficits in attention deficit hyperactivity disorder. Sci. Rep. 10:529. doi: 10.1038/s41598-019-56936-4, PMID: 31953449 PMC6969149

[ref99] Maresca G. Leonardi S. De Cola M. C. Giliberto S. Di Cara M. Corallo F.. (2022). Use of virtual reality in children with dyslexia. Children 9:1621. doi: 10.3390/children9111621, PMID: 36360349 PMC9688381

[ref100] Martínez-Briones B. J. Fernández-Harmony T. Gómez N. G. Biscay-Lirio R. Bosch-Bayard J. (2020). Working memory in children with learning disorders: an EEG power spectrum analysis. Brain Sci. 10:817. doi: 10.3390/brainsci10110817, PMID: 33158135 PMC7694181

[ref101] Merzon L. Pettersson K. Aronen E. T. Huhdanpää H. Seesjärvi E. Henriksson L.. (2022). Eye movement behavior in a real-world virtual reality task reveals ADHD in children. Sci. Rep. 12:20308. doi: 10.1038/s41598-022-24552-4, PMID: 36434040 PMC9700686

[ref102] Mesa-Gresa P. Gil-Gómez H. Lozano-Quilis J. Gil-Gómez J. (2018). Effectiveness of virtual reality for children and adolescents with autism spectrum disorder: an evidence-based systematic review. Sensors 18:2486. doi: 10.3390/s18082486, PMID: 30071588 PMC6111797

[ref103] Miller I. Wiederhold B. K. Miller C. S. Wiederhold M. D. (2020). Virtual reality air travel training with children on the autism spectrum: a preliminary report. Cyberpsychol. Behav. Soc. Netw. 23, 10–15. doi: 10.1089/cyber.2019.0093, PMID: 31355673

[ref104] Miyake A. Friedman N. P. Emerson M. J. Witzki A. Howerter A. Wager T. D. (2000). The unity and diversity of executive functions and their contributions to complex “frontal lobe” tasks: a latent variable analysis. Cogn. Psychol. 41, 49–100. doi: 10.1006/cogp.1999.0734, PMID: 10945922

[ref105] Mokobane M. Pillay B. Meyer A. (2019). Fine motor deficits and attention deficit hyperactivity disorder in primary school children. S. Afr. J. Psychiatry 25:1232. doi: 10.4102/sajpsychiatry.v25i0.1232, PMID: 30899581 PMC6424539

[ref106] Moll K. Kunze S. Neuhoff N. Bruder J. Schulte-Körne G. (2014). Specific learning disorder: prevalence and gender differences. PLoS One 9:e103537. doi: 10.1371/journal.pone.0103537, PMID: 25072465 PMC4114805

[ref108] Nachshon O. Horowitz-Kraus T. (2018). Cognitive and emotional challenges in children with reading difficulties. Acta Paediatr. 108, 1110–1114. doi: 10.1111/apa.14672, PMID: 30506734 PMC6521714

[ref109] Nigg J. T. Blaskey L. Stawicki J. A. Sachek J. (2004). Evaluating the endophenotype model of ADHD neuropsychological deficit: results for parents and siblings of children with ADHD combined and inattentive subtypes. J. Abnorm. Psychol. 113, 614–625. doi: 10.1037/0021-843x.113.4.614, PMID: 15535793

[ref110] North M. M. North S. Coble J. K. (1998). Virtual reality therapy: an effective treatment for phobias. Stud. Health Technol. Inform 58, 112–119. Available at: https://pubmed.ncbi.nlm.nih.gov/10350911 10350911

[ref111] Orsolini M. Federico F. Vecchione M. Pinna G. Capobianco M. Melogno S. (2023). How is working memory related to reading comprehension in Italian monolingual and bilingual children? Brain Sci. 13:58. doi: 10.3390/brainsci13010058, PMID: 36672040 PMC9856821

[ref112] Pang X. Wang H. Dill S. Boswell M. Pang X. Singh M. K.. (2021). Attention deficit hyperactivity disorder (ADHD) among elementary students in rural China: prevalence, correlates, and consequences. J. Affect. Disord. 293, 484–491. doi: 10.1016/j.jad.2021.06.014, PMID: 34280772

[ref113] Parsons T. D. Bowerly T. Buckwalter J. G. Rizzo A. A. (2007). A controlled clinical comparison of attention performance in children with ADHD in a virtual reality classroom compared to standard neuropsychological methods. Child Neuropsychol. 13, 363–381. doi: 10.1080/13825580600943473, PMID: 17564852

[ref114] Parsons T. D. Duffield T. Asbee J. (2019). A comparison of virtual reality classroom continuous performance tests to traditional continuous performance tests in delineating ADHD: a meta-analysis. Neuropsychol. Rev. 29, 338–356. doi: 10.1007/s11065-019-09407-6, PMID: 31161465

[ref115] Parsons T. D. Riva G. Parsons S. Mantovani F. Newbutt N. Lin L.. (2017). Virtual reality in pediatric psychology. Pediatrics 140, S86–S91. doi: 10.1542/peds.2016-1758i, PMID: 29093039

[ref116] Polanczyk G. De Lima M. S. Horta B. L. Biederman J. Rohde L. A. (2007). The worldwide prevalence of ADHD: a systematic review and metaregression analysis. Am J. Psychiatry 164, 942–948. doi: 10.1176/ajp.2007.164.6.942, PMID: 17541055

[ref117] Pollak Y. Weiss P. L. Rizzo A. A. Weizer M. Shriki L. Shalev R. S.. (2009). The utility of a continuous performance test embedded in virtual reality in measuring ADHD-related deficits. J. Dev. Behav. Pediatr. 30, 2–6. doi: 10.1097/dbp.0b013e3181969b22, PMID: 19194324

[ref118] Ramsey K. A. Essoe J. K. Boyle N. Patrick A. K. McGuire J. F. (2023). Immersive virtual reality exposures for the treatment of childhood anxiety. Child Psychiatry Hum. Dev. doi: 10.1007/s10578-023-01628-4, PMID: 37985621

[ref119] Richards G. Samuels S. J. Turnure J. E. Ysseldyke J. E. (1990). Sustained and selective attention in children with learning disabilities. J. Learn. Disabil. 23, 129–136. doi: 10.1177/002221949002300210 2303740

[ref120] Riva G. Davide F. IJsselsteijn W. A. (2003). *Being there: concepts, effects and measurements of user presence in synthetic environments*. Available at: https://psycnet.apa.org/record/2003-07184-000

[ref121] Rodrigo-Yanguas M. Martin-Moratinos M. Menendez-Garcia A. Gonzalez-Tardon C. Royuela A. Blasco-Fontecilla H. (2021). A virtual reality game (the Secret Trail of moon) for treating attention-deficit/hyperactivity disorder: development and usability study. JMIR Serious Games 9:e26824. doi: 10.2196/26824, PMID: 34468332 PMC8444038

[ref122] Romero-Ayuso D. Toledano-González A. Del Carmen Rodríguez-Martínez M. Arroyo-Castillo P. Triviño-Juárez J. M. González P.. (2021). Effectiveness of virtual reality-based interventions for children and adolescents with ADHD: a systematic review and meta-analysis. Children 8:70. doi: 10.3390/children8020070, PMID: 33494272 PMC7909839

[ref123] Rylaarsdam L. Guemez-Gamboa A. (2019). Genetic causes and modifiers of autism spectrum disorder. Front. Cell. Neurosci. 13:385. doi: 10.3389/fncel.2019.00385, PMID: 31481879 PMC6710438

[ref124] Sahoo M. K. Biswas H. Padhy S. K. (2015). Psychological co-morbidity in children with specific learning disorders. J. Family Med. Prim. Care 4, 21–25. doi: 10.4103/2249-4863.152243, PMID: 25810984 PMC4367000

[ref125] Salari N. Ghasemi H. Abdoli N. Rahmani A. Shiri M. H. Hashemian A. H.. (2023). The global prevalence of ADHD in children and adolescents: a systematic review and meta-analysis. Ital. J. Pediatr. 49:48. doi: 10.1186/s13052-023-01456-1, PMID: 37081447 PMC10120242

[ref126] Satu P. Minna L. Satu S. (2023). Immersive VR assessment and intervention research of individuals with neurodevelopmental disorders is dominated by ASD and ADHD: a scoping review. Rev. J. Autism Dev. Disord. doi: 10.1007/s40489-023-00377-3

[ref127] Schueller S. M. Stiles-Shields C. Yarosh L. (2017). Online treatment and virtual therapists in child and adolescent psychiatry. Child Adolesc. Psychiatr. Clin. N. Am. 26, 1–12. doi: 10.1016/j.chc.2016.07.011, PMID: 27837935 PMC5123797

[ref128] Seesjärvi E. Puhakka J. Aronen E. T. Lipsanen J. Mannerkoski M. Hering A.. (2021). Quantifying ADHD symptoms in open-ended everyday life contexts with a new virtual reality task. J. Atten. Disord. 26, 1394–1411. doi: 10.1177/10870547211044214, PMID: 34865551 PMC9304743

[ref129] Shahmoradi L. Rezayi S. (2022). Cognitive rehabilitation in people with autism spectrum disorder: a systematic review of emerging virtual reality-based approaches. J. Neuroeng. Rehabil. 19:91. doi: 10.1186/s12984-022-01069-5, PMID: 35982460 PMC9389666

[ref130] Shema-Shiratzky S. Brozgol M. Cornejo-Thumm P. Geva-Dayan K. Rotstein M. Leitner Y.. (2018). Virtual reality training to enhance behavior and cognitive function among children with attention-deficit/hyperactivity disorder: brief report. Dev. Neurorehabil. 22, 431–436. doi: 10.1080/17518423.2018.1476602, PMID: 29771624

[ref131] Shiri S. Tenenbaum A. Sapir-Budnero O. Wexler I. D. (2014). Elevating hope among children with attention deficit and hyperactivity disorder through virtual reality. Front. Hum. Neurosci. 8:198. doi: 10.3389/fnhum.2014.00198, PMID: 24847233 PMC4019862

[ref132] Skalski S. Konaszewski K. Pochwatko G. Balas R. Surzykiewicz J. (2021). Effects of hemoencephalographic biofeedback with virtual reality on selected aspects of attention in children with ADHD. Int. J. Psychophysiol. 170, 59–66. doi: 10.1016/j.ijpsycho.2021.10.001, PMID: 34653532

[ref133] So B. P. Lai D. K. Cheung D. S. K. Lam W. Cheung J. C. Wong D. W. (2022). Virtual reality-based immersive rehabilitation for cognitive- and behavioral-impairment-related eating disorders: A VREHAB framework scoping review. Int. J. Environ. Res. Public Health 19:5821. doi: 10.3390/ijerph19105821, PMID: 35627357 PMC9141870

[ref134] Solan H. A. (1993). Dyslexia and learning disabilities. Optom. Vis. Sci. 70, 343–347. doi: 10.1097/00006324-199305000-00001, PMID: 8515960

[ref135] Steiner N. J. Frenette E. C. Rene K. M. Brennan R. T. Perrin E. C. (2014). In-school neurofeedback training for ADHD: sustained improvements from a randomized control trial. Pediatrics 133, 483–492. doi: 10.1542/peds.2013-2059, PMID: 24534402

[ref136] Stokes J. D. Rizzo A. Geng J. J. Schweitzer J. B. (2022). Measuring attentional distraction in children with ADHD using virtual reality technology with eye-tracking. Front. Virtual Real. 3:855895. doi: 10.3389/frvir.2022.855895, PMID: 35601272 PMC9119405

[ref137] Su Y. Cheng H. Lai C. (2022). Study of virtual reality immersive technology enhanced mathematics geometry learning. Front. Psychol. 13:760418. doi: 10.3389/fpsyg.2022.760418, PMID: 35250708 PMC8892099

[ref138] Tan B. Shi J. Yang S. Loh H. Ng D. Choo C.. (2022). The use of virtual reality and augmented reality in psychosocial rehabilitation for adults with neurodevelopmental disorders: A systematic review. Front. Psych. 13:1055204. doi: 10.3389/fpsyt.2022.1055204, PMID: 36590624 PMC9794993

[ref139] Thomas R. Sanders S. Doust J. Beller E. Glasziou P. (2015). Prevalence of attention-deficit/hyperactivity disorder: A systematic review and meta-analysis. Pediatrics 135, e994–e1001. doi: 10.1542/peds.2014-3482 25733754

[ref140] Toffalini E. Marsura M. Garcia R. B. Cornoldi C. (2018). A cross-modal working memory binding span deficit in reading disability. J. Learn. Disabil. 52, 99–108. doi: 10.1177/0022219418786691, PMID: 29985098

[ref141] Tosto M. G. Momi S. K. Asherson P. Malki K. (2015). A systematic review of attention deficit hyperactivity disorder (ADHD) and mathematical ability: current findings and future implications. BMC Med. 13:204. doi: 10.1186/s12916-015-0414-4, PMID: 26315934 PMC4552374

[ref142] Valentine A. Brown B. J. Groom M. J. Young E. Hollis C. Hall C. L. (2020). A systematic review evaluating the implementation of technologies to assess, monitor and treat neurodevelopmental disorders: A map of the current evidence. Clin. Psychol. Rev. 80:101870. doi: 10.1016/j.cpr.2020.101870, PMID: 32712216

[ref143] Van Der Oord S. Ponsioen A. Geurts H. M. Brink E. L. T. Prins P. J. M. (2012). A pilot study of the efficacy of a computerized executive functioning remediation training with game elements for children with ADHD in an outpatient setting. J. Atten. Disord. 18, 699–712. doi: 10.1177/1087054712453167, PMID: 22879577

[ref144] Vasileva M. Graf R. K. Reinelt T. Petermann U. Petermann F. (2020). Research review: A meta-analysis of the international prevalence and comorbidity of mental disorders in children between 1 and 7 years. J. Child Psychol. Psychiatry Allied Discip. 62, 372–381. doi: 10.1111/jcpp.13261, PMID: 32433792

[ref145] Wang M. Reid D. (2010). Virtual reality in pediatric neurorehabilitation: attention deficit hyperactivity disorder, autism and cerebral palsy. Neuroepidemiology 36, 2–18. doi: 10.1159/000320847, PMID: 21088430

[ref146] Wiguna T. Bahana R. Dirgantoro B. Minayati K. Teh S. D. Ismail R. I.. (2022). Developing attention deficits/hyperactivity disorder-virtual reality diagnostic tool with machine learning for children and adolescents. Front. Psych. 13:984481. doi: 10.3389/fpsyt.2022.984481, PMID: 36213908 PMC9533640

[ref147] Wiguna T. Wigantara N. A. Ismail R. I. Kaligis F. Minayati K. Bahana R.. (2020). A four-step method for the development of an ADHD-VR digital game diagnostic tool prototype for children using a DL model. Front. Psych. 11:829. doi: 10.3389/fpsyt.2020.00829, PMID: 32973578 PMC7461963

[ref148] Willcutt E. G. (2012). The prevalence of DSM-IV attention-deficit/hyperactivity disorder: A Meta-analytic review. Neurotherapeutics 9, 490–499. doi: 10.1007/s13311-012-0135-8, PMID: 22976615 PMC3441936

[ref149] Wong K. P. Qin J. (2023). Effectiveness of social virtual reality training in enhancing social interaction skills in children with attention-deficit/hyperactivity disorder: protocol for a three-arm pilot randomized controlled trial. JMIR Res. Protocol. 12:e48208. doi: 10.2196/48208, PMID: 37721790 PMC10546265

[ref150] Wong K. P. Qin J. Xie Y. J. Zhang B. (2023). Effectiveness of technology-based interventions for school-age children with attention-deficit/hyperactivity disorder: systematic review and meta-analysis of randomized controlled trials. JMIR Mental Health 10:e51459. doi: 10.2196/51459, PMID: 37988139 PMC10698651

[ref151] Xie S. Karlsson H. Dalman C. Widman L. Rai D. Gardner R. M.. (2020). The familial risk of autism spectrum disorder with and without intellectual disability. Autism Res. 13, 2242–2250. doi: 10.1002/aur.2417, PMID: 33103358 PMC7821228

[ref152] Yeh S. Lin S. Wu E. H. Zhang K. Xiu X. Rizzo A.. (2020). A virtual-reality system integrated with neuro-behavior sensing for attention-deficit/hyperactivity disorder intelligent assessment. IEEE Trans. Neural Syst. Rehabil. Eng. 28, 1899–1907. doi: 10.1109/tnsre.2020.3004545, PMID: 32746303

[ref153] Yip B. Man D. (2009). Virtual reality (VR)-based community living skills training for people with acquired brain injury: A pilot study. Brain Inj. 23, 1017–1026. doi: 10.3109/02699050903379412, PMID: 19891532

[ref154] Yuan S. N. V. Ip H. H. S. (2018). Using virtual reality to train emotional and social skills in children with autism spectrum disorder. London J. Prim. Care 10, 110–112. doi: 10.1080/17571472.2018.1483000, PMID: 30083244 PMC6074644

[ref155] Zhang F. Zhang Y. Li G. Luo H. (2023). Using virtual reality interventions to promote social and emotional learning for children and adolescents: a systematic review and meta-analysis. Children 11:41. doi: 10.3390/children11010041, PMID: 38255355 PMC10813885

[ref156] Zhao X. Page T. F. Altszuler A. R. Pelham W. E. Kipp H. Gnagy E. M.. (2019). Family burden of raising a child with ADHD. J. Abnorm. Child Psychol. 47, 1327–1338. doi: 10.1007/s10802-019-00518-5, PMID: 30796648

[ref157] Zhao J. Zhang X. Lu Y. Wu X. Zhou F. Shi-Chang Y.. (2022). Virtual reality technology enhances the cognitive and social communication of children with autism spectrum disorder. Front. Public Health 10:1029392. doi: 10.3389/fpubh.2022.1029392, PMID: 36276341 PMC9582941

